# Methylene Blue in Pulmonary Tuberculosis

**Published:** 1901-10

**Authors:** 


### Methylene Blue in Pulmonary Tuberculosis.

Cornelius Lane, M. D., *American Medicine,* September 7th, reports that he has obtained excellent results from the use of one grain doses of methylene blue three times a day in pulmonary tuberculosis. The drug seems to control the temperature and pulse and to improve the appetite.

